# Supplementary material for: Genome-wide functional screening of drug-resistance genes in Plasmodium falciparum
Source: Nat Commun. 2022 Oct 18;13:6163. doi: 10.1038/s41467-022-33804-w (PMC9579134; doi:10.1038/s41467-022-33804-w)
Supplement: Supplementary file 1 — Supplementary information [file 41467_2022_33804_MOESM1_ESM.pdf]

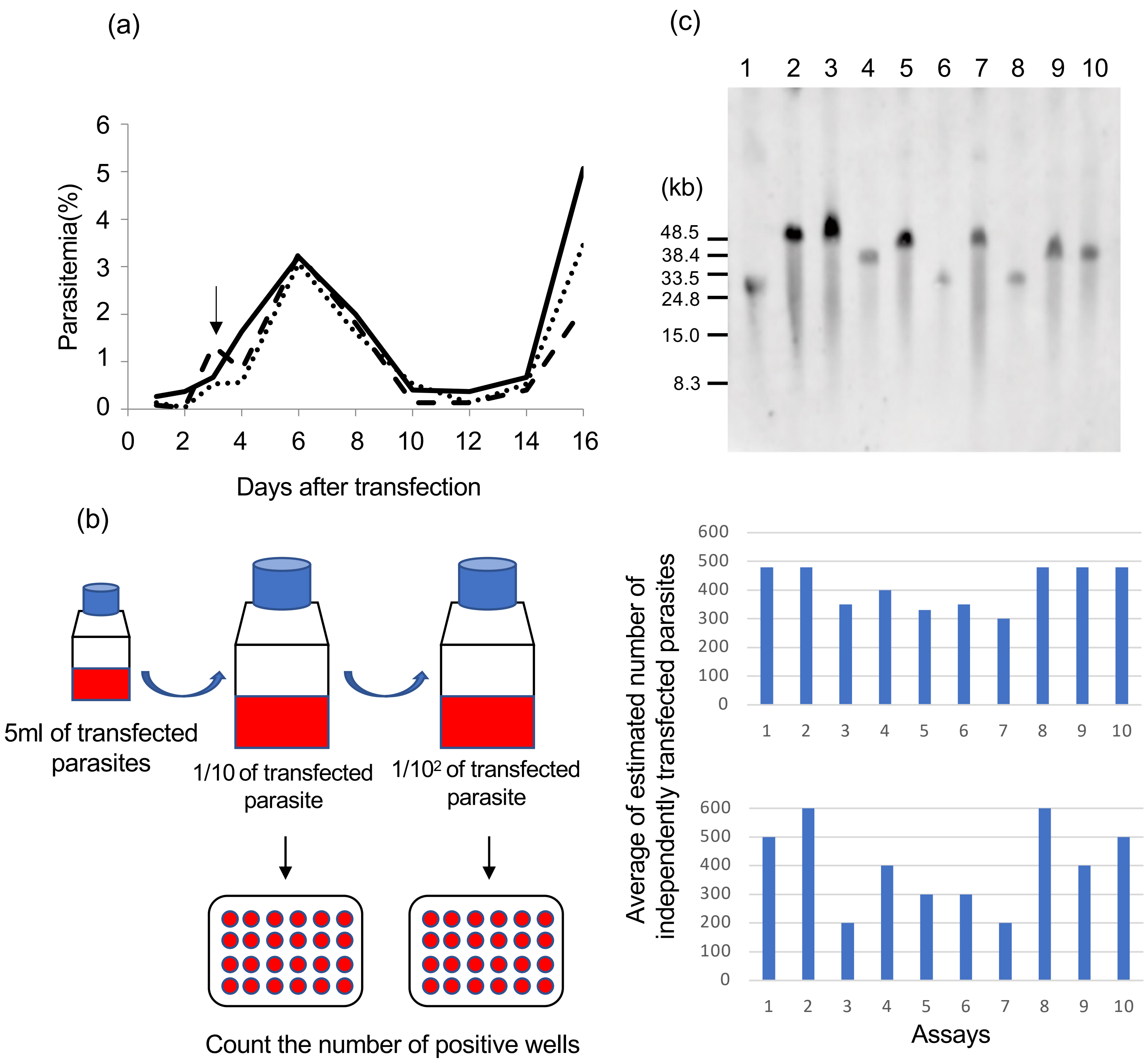

Supplementary Figure 1| Genomic library construction in *P. falciparum*. (a) Five micrograms of partially digested genomic DNA fragments was ligated to *Bam*HI-digested pFCENv1 and was then introduced into parasites. The parasitemia was monitored in three independent transfection experiments. The numbers of independently transfected parasites were estimated at 702.3 (solid line), 561.8 (dotted line) and 421.4 (dashed line) in each transfection experiment. The arrow indicates the initiation of the pyrimethamine treatment used to establish the genomic library. (b) The culture containing parasites were diluted immediately after transfection; all diluted culture were dispensed into 48 wells; the parasite in wells were cultured more the 3 weeks. After long-term culture, the number of the parasite-positive wells were counted. The number of independently transfected parasites were estimated by 10 assays. Upper and lower graphs were the results obtained using 1/10 and 10^2 of transfected parasites, respectively. Source data are provided as a Source Data file. (c) Ten clonal parasites (lanes 1 - 10) were randomly selected from the genomic library, which was generated by one transfection using 5 μg of DNA fragments. The DNA fragments inserted in pFCENv1 were analyzed by CHEF electrophoresis, followed by Southern blot hybridization using the *hdhfr* gene as the DNA probe.

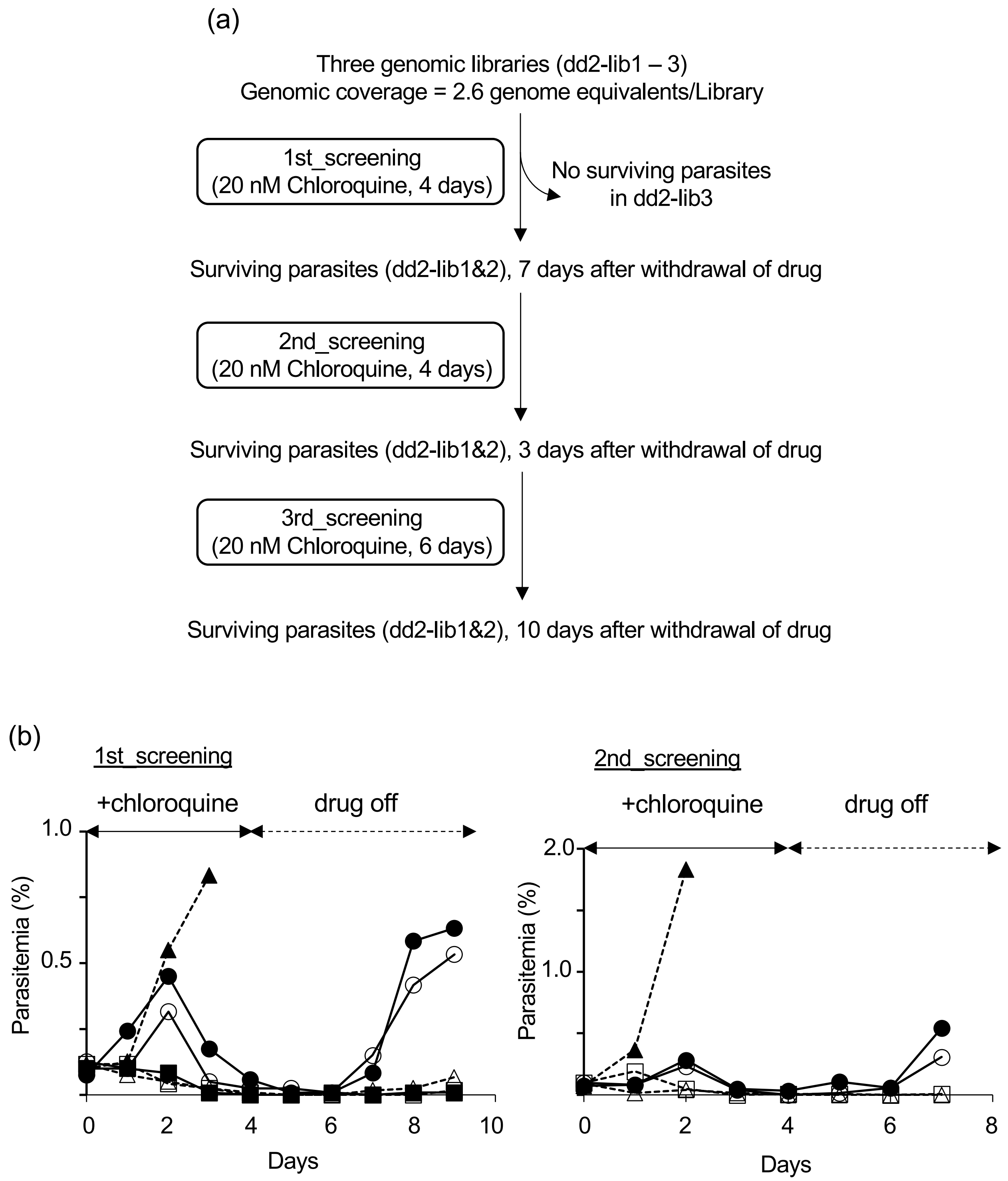

Supplementary Figure 2| Functional screening of the chloroquine resistance gene. (a). Schematic of the functional screening used to identify chloroquine resistance genes from the genomic libraries, dd2-lib1 - 3. (b). The parasites in those genomic libraries were treated with 20 nM chloroquine for four days (1st_ screening). Parasite survival in each library was monitored every 24 hours, and parasites were detected in two libraries (dd2-lib1 and 2). Surviving parasites were further treated as in the 1st_screening (2nd_screening). Surviving parasites after two rounds of drug screening were treated under harsher conditions, as shown in (a). The parasitemia levels in dd2-lib1, 2, and 3 are indicated as closed circles, open circles, and closed squares in solid lines, respectively. In addition, those of strains 3D7 and Dd2 and the negative control parasites are shown as open triangles, closed triangles, and open squares in dashed lines. Source data are provided as a Source Data file.

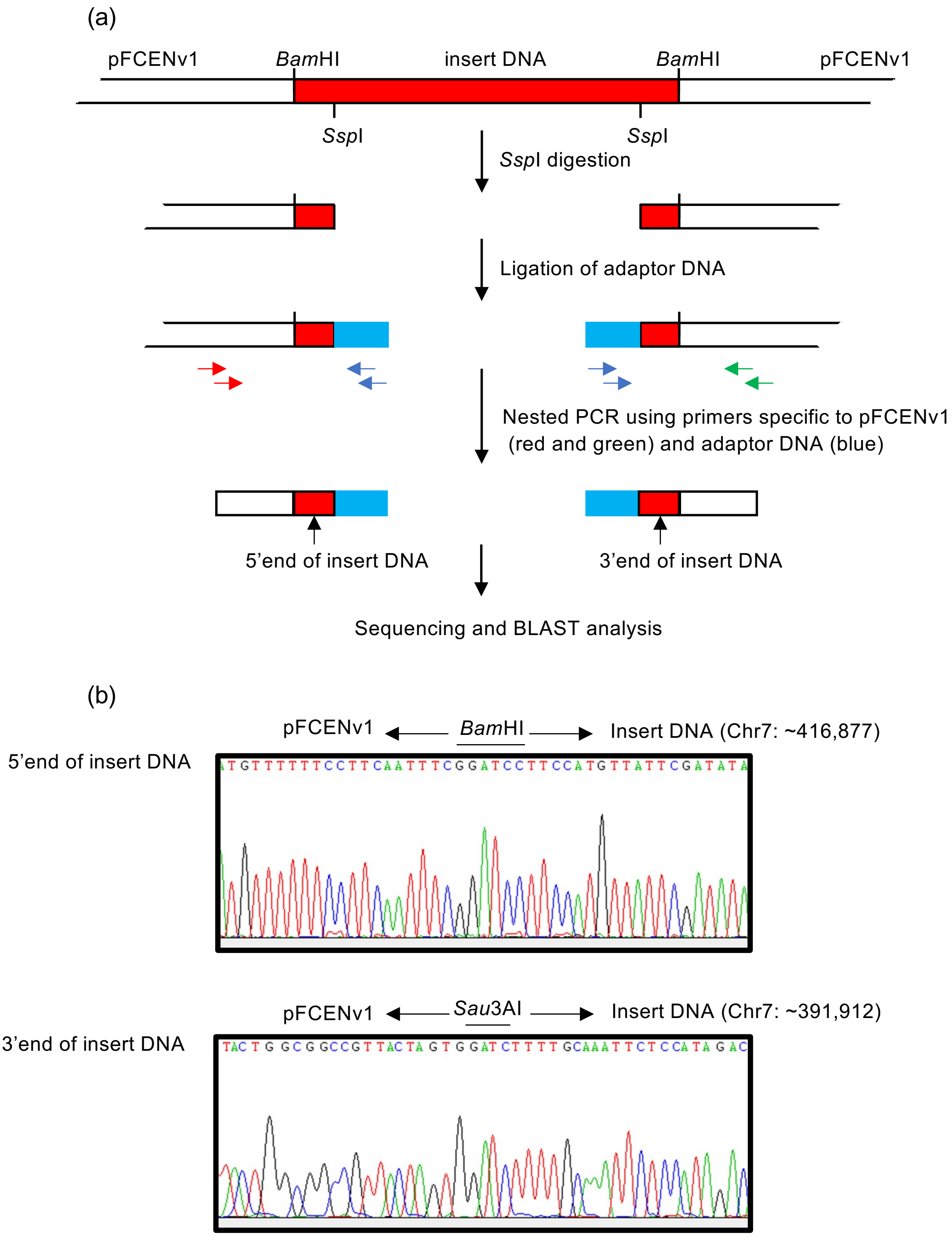

Supplementary Figure 3| Sequence analysis of insert DNA in pFCENv1 recovered from selected chloroquine-resistant parasites. (a). A schematic of the sequence analysis of insert DNA in pFCENv1 was shown. The amplified PCR products encoded both the 5'- and 3'-ends of the insert DNA in addition to the pFCENv1 and adaptor DNA. The genomic position of the insert DNA was identified by sequence identity searches using the BLAST program with the obtained sequences from the 5'- and 3'-ends of the insert DNA (b). Raw peak data for the 5'- and 3'-ends of the insert DNA fragment, which was recovered from the parasite clone selected from dd2-1ib2, are shown. The insert DNA was cloned into the *Bam*HI site of pFCENv1. The amplified PCR products contained the ends of the insert DNA and pFCENv1, as described above.

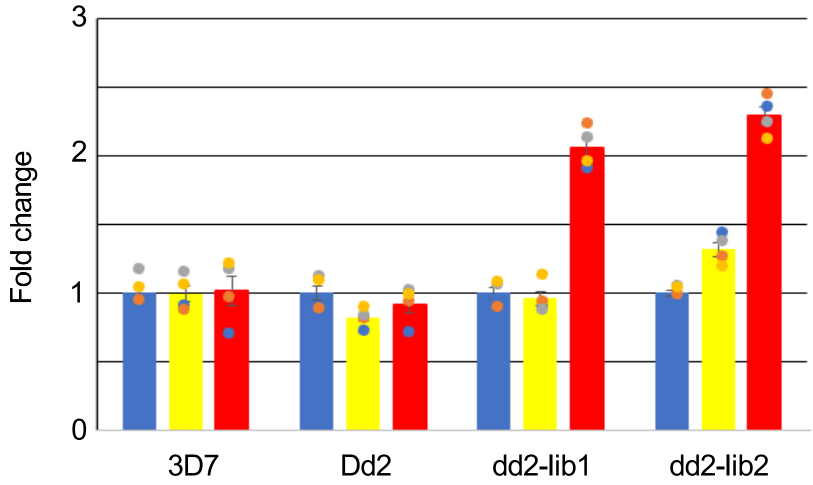

Supplementary Figure 4| Copy number analysis of *pfcrt* of parasite clones, which were selected from libraries by chloroquine screening. The pFCENv1 with insert DNA was purified from parasite clones, which were selected from dd2-lib1 and -2, along with genomic DNA of the parasites. The copy numbers of *pfcrt* on the recovered pFCENv1 were estimated by quantitative PCR using primers specific to the *pfcrt* gene (red), as described in the Materials and Methods. The β-tubulin gene (blue) was used as a single copy reference gene. In addition, the fructose-bisphosphate aldolase gene (yellow), which was the single copy gene, was also used as a reference. Assays were performed using n=4 biological independent samples. Error bars are SEM, and measure of the center is the means value. Source data are provided as a Source Data file.

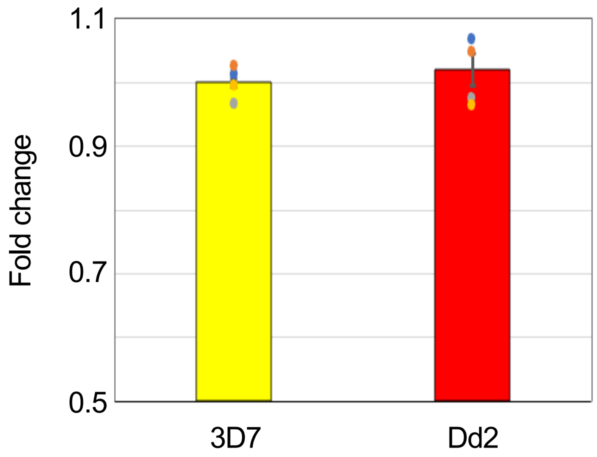

Supplementary Figure 5| quantitative analysis of expression of *pfcrt*. The mRNA level of *pfcrt* in those strains were analyzed using RT-qPCR using mRNA purified from strains 3D7 (yellow) and Dd2 (red). in biological independent quadruple assays. The seryl-tRNA gene were used as internal control. The assays were performed using n=4 biological independent samples. Error bar shows SEM, and measure of the center is the means value. Source data are provided as a Source Data file.

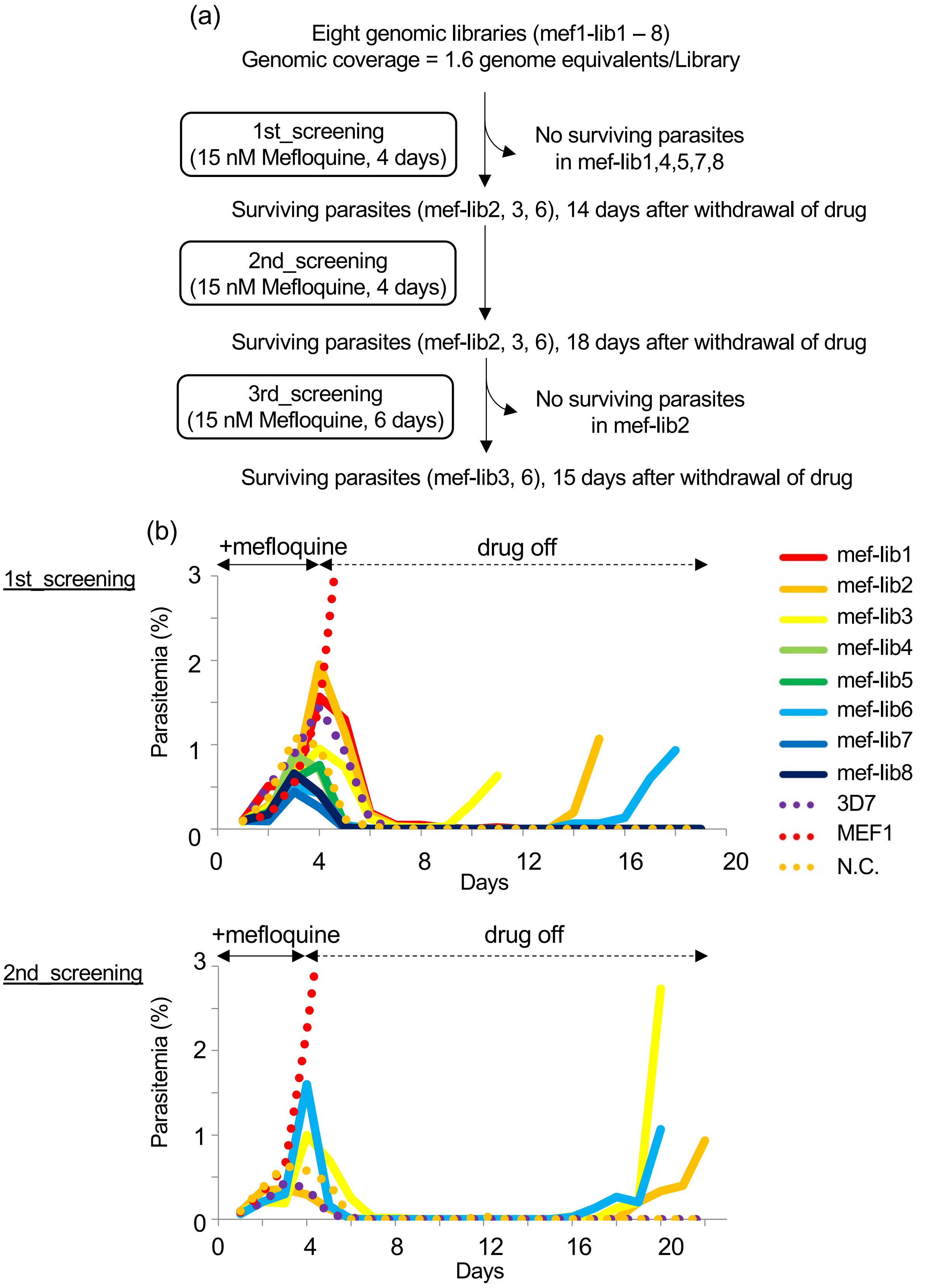

Supplementary Figure 6| Functional screening of the mefloquine resistance gene. (a). Schematic of functional screening to identify mefloquine resistance genes from genomic libraries (mef-lib1 - 8). (b). The parasites in each genomic library were treated with 15 nM mefloquine for four days, and their survival was monitored (1st_screening). The surviving parasites were then treated with mefloquine, as in the 1st_screening (2nd_screening). Parasite survival was detected in mef-lib2, 3, and 6, and they were again treated with mefloquine. The legends for all parasites are indicated at the right side of the figure. Source data are provided as a Source Data file.

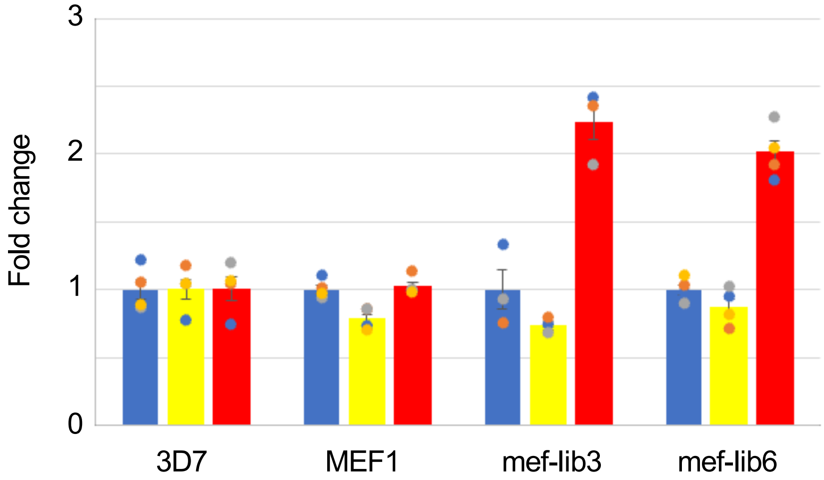

Supplementary Figure 7| Copy number analysis of pFCENv1 recovered from parasite clones, which were selected from libraries by mefloquine screening. The copy numbers of the recovered pFCENv1 were estimated by quantitative PCR using primers specific to the *pfmdr7* gene (red), as described in the Materials and Methods. The β-tubulin gene (blue) was used as the single copy reference gene. In addition, the fructose-bisphosphate aldolase gene (red), which was a single copy gene, was used similarly as an internal reference as in Supplementary Figure S4. SEM were indicated as error bars. The measure of the center is the means value. Assays were performed independently in quadruple. Source data are provided as a Source Data file.

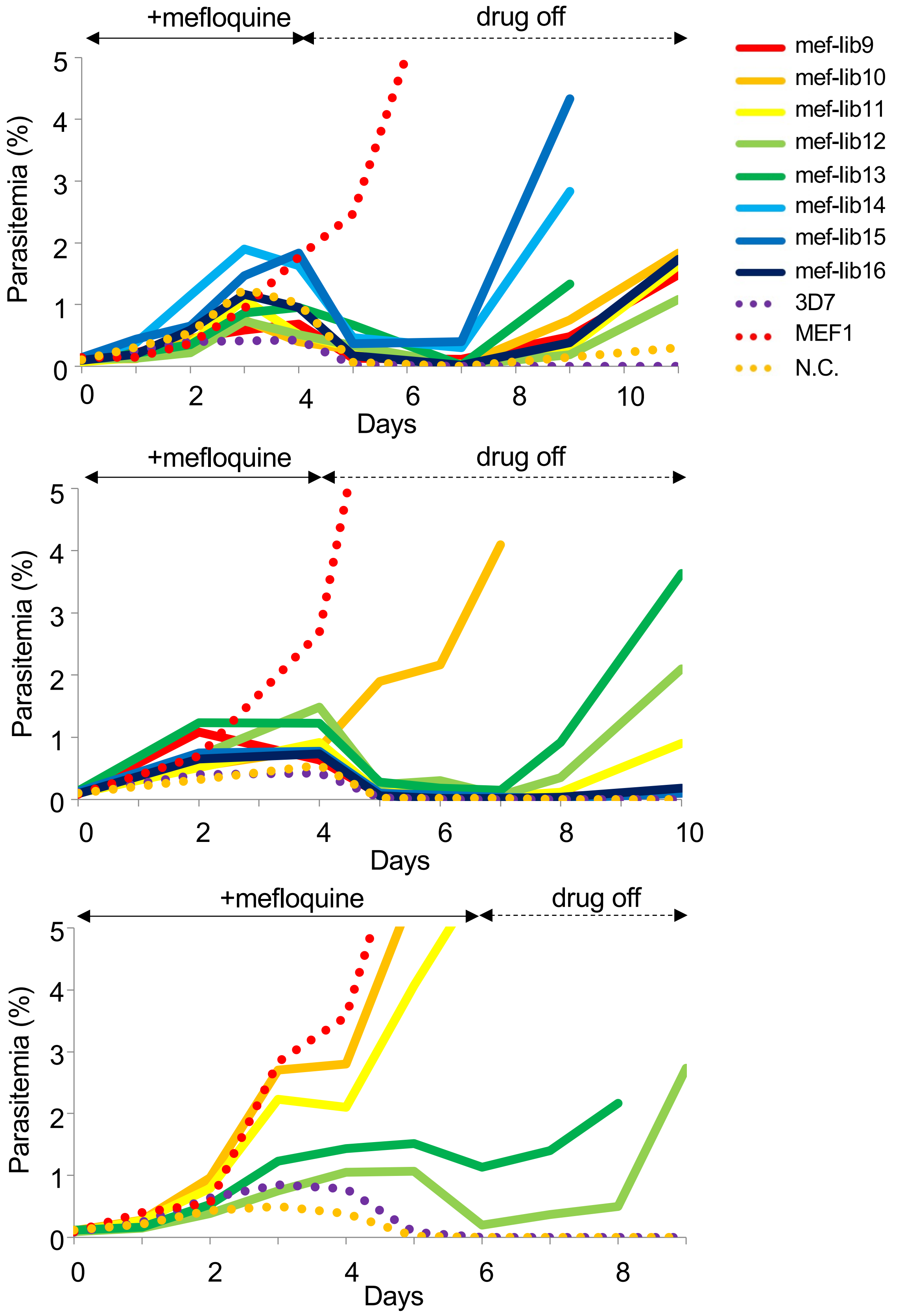

Supplementary Figure 8| Functional screening to identify mefloquine resistance genes. Eight additional genomic libraries (mef-lib9 - 16) were generated from strain MEF1. Parasites that newly acquired mefloquine resistance were selected by three rounds of drug screening. The legends for all samples are indicated in the figure. Source data are provided as a Source Data file.

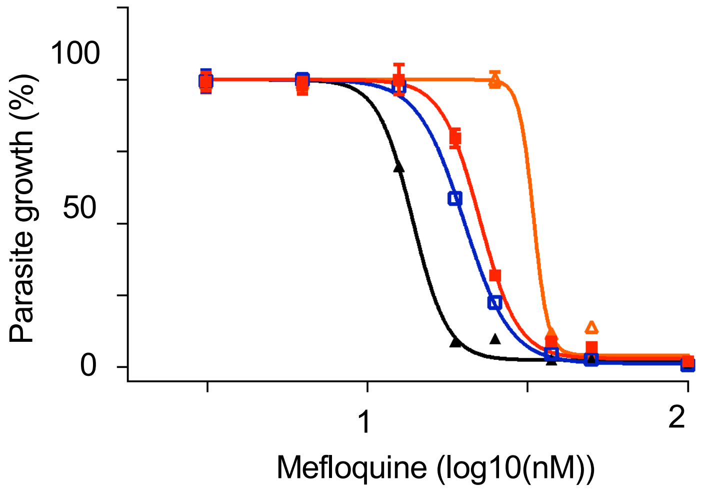

Supplementary Figure 9| Assay for estimating the IC_{50} values of the transgenic parasites. The transgenic parasites, in which the *pfmdr7* of the MEF1 (red) and the3D7 (blue) were introduced using the pFCENv1 and their IC_{50} values were determined. In addition, those of the 3D7 (black) and the MEF1 (orange) were also determined. SEM were indicated as error bars. All assay were performed independently quadruplicate. Source data are provided as a Source Data file.

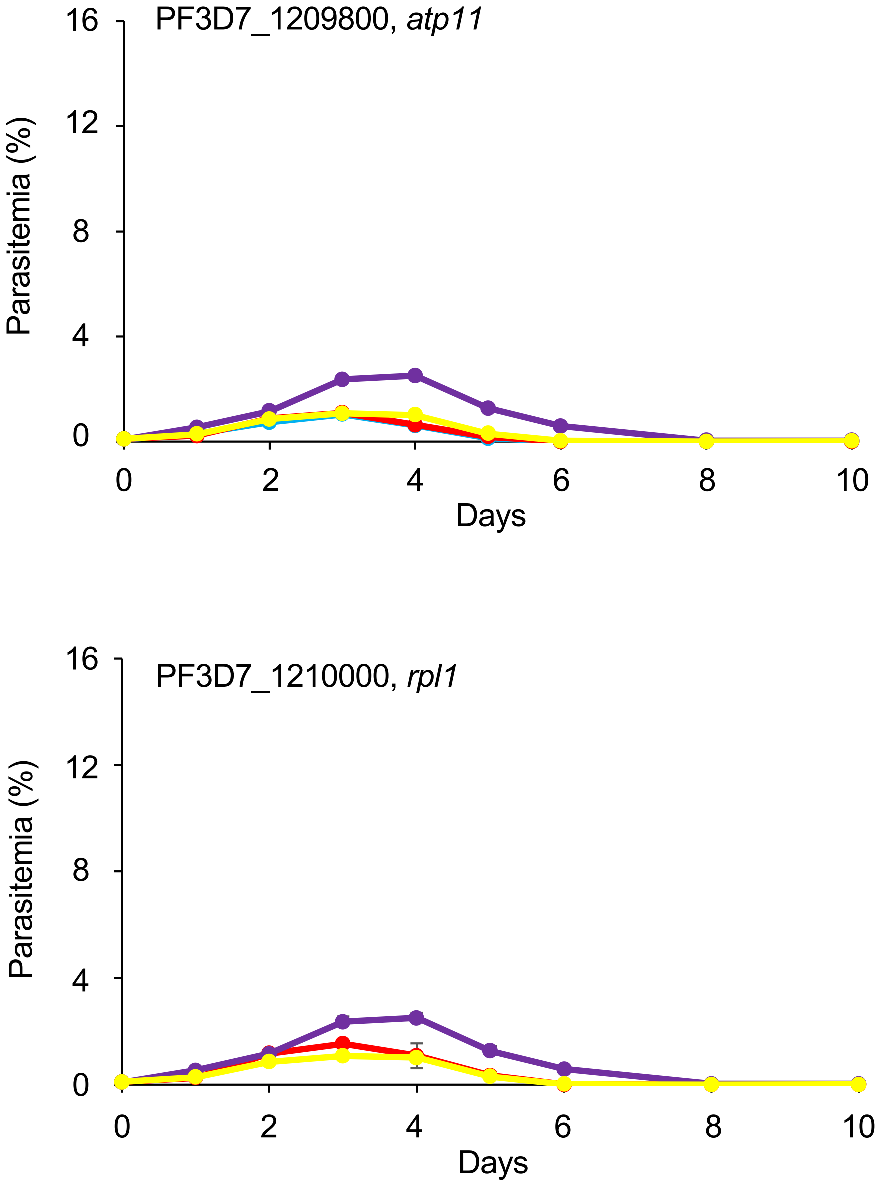

Supplementary Figure 10| Functional analysis of PfMDR7. The *atp11* and *rpl1* genes of strains MEF1 and 3D7 were introduced into drug-sensitive parasites, *i.e.,* strain 3D7. The resulting transgenic parasites were continuously treated with 15 nM drug for six days and were subsequently cultured in the absence of drug. The mefloquine resistance of those transgenic parasites were evaluated based on their survival. The parasitemia of the transgenic parasites in which two genes of strains MEF1 and 3D7 were introduced are indicated in red and blue, respectively, in each graph. Those of strain 3D7 and the negative control parasites are indicated with purple and yellow, respectively. The assays were performed using two biological independent transgenic parasites, in which *atp11* and *rpl1* genes were introduced. Error bars are SEM, and measure of center is the means value. Source data are provided as a Source Data file.

```
pfmdr7_3d7      TAATGTTATAGTTTCAAAAAAAAAAAAAAAAAAAAAAAAAAAACTTGTACAACATATAT 60
pfmdr7_mef1     TAATGTTATAGTTTCAA----AAAAAAAAAAAAAAAAAAAAAAACTTGTACAACACATAT 56
                *****************    ********************************** ****

pfmdr7_3d7      ATTAATATATTTTTTTGTCATATATAATATATATCCATACAATAATACTTTATTAGAATA 120
pfmdr7_mef1     ATTAATATATTTTTTTGTCATATATAATATATATCCATACAATAATACTTTATTAAAATA 116
                ******************************************************* ****

pfmdr7_3d7      TTAGTTCATCAATTAAAAGGATAAAAAAAAAATTAAGAAAAATAAACTTATGGATTGTCT 180
pfmdr7_mef1     TTAGTTCATCAATTAAAAGGATAAAAAAAAAATTAAGGAAAATAAACTTATGGATTGTCT 176
                ************************************* **********************

pfmdr7_3d7      TCTAAATTGTGTTTTTTTTTTTTTTTTTTTTTTTTTTAATTCCCAAATATAAATAATATAT 240
pfmdr7_mef1     TCTAAATTGGGTTTTTTTTTTT---TTTTTTTTTTTAATTCCCAAATATAAATAATATAT 233
                ********* ************   ***********************************

pfmdr7_3d7      GTAAATGAAAAATATATATATATATATATTATATATATATCATCATATATATGTGCTTTA 300
pfmdr7_mef1     GTAAATGAAAAATATATATATATATATATTATATATATATCATCATATATATGTGCTTTA 293
                ************************************************************

pfmdr7_3d7      TTTTTTTTCTTTTCTTTTTTTTTGGTATAATTATATTATTGTTCCTGAGAATTTTAATTA 360
pfmdr7_mef1     TTTTTTTTCTTTTCTTTTTTTTTGGTATAATTATATTATTGTTCCTGAGAATTTTAATTA 353
                ************************************************************

pfmdr7_3d7      TAGGAAAAAAATGTTTTCCTGCCATCCGCTAAAATAAATTATATAATATGATATATTATA 420
pfmdr7_mef1     TAGGAAA-AAATGTTTTCCTGCCATCCGATAAAATAAATTATATAATATGATATATTATA 412
                ******* ******************** *******************************

pfmdr7_3d7      TTTCGCCCTTAGTAAATATTGTATAATATTTATTATAATTTATATATGTATATATATTTT 480
pfmdr7_mef1     TTTCGCCCTTAGTAAATATTGTATAATATTTATTATAATTTATATATGTATATATATTTT 472
                ************************************************************

pfmdr7_3d7      TCTTTTGGGGGGTAAAATTAAAAAATAGAAGTAATTAATTTTTTTCTCCCAGAATAAAAA 540
pfmdr7_mef1     TCTTTTGGGGGGTAAAATTAAAAAATAGAAGTAATTAATTTTTTTCTCCCAGAATAAAAA 532
                ************************************************************

pfmdr7_3d7      ATTGATATGCATAAAAAGAGGTACACATAAAATATATATATATGTATATATTTATTTATT 600
pfmdr7_mef1     ATTGATATGCATAAAAAGAGGTACACATAAAATATATATATATGTATATATTTATTTATT 592
                ************************************************************

pfmdr7_3d7      TATCATGGCAGATATATAATTTTTTTTTTTTTTTTTTTTCTTTTTTTAATTTCATATACA 660
pfmdr7_mef1     TATCATGGCAGATATATAATTTTTTTTTTTT-TTTTTTCTTTTTTTAATTTCATATACA 651
                ******************************** ***************************

pfmdr7_3d7      ACATAATACATACGTAGCTAGCGTCGAG 688
pfmdr7_mef1     ACATAATACATACGTAGCTAGCGTCGAG 679
                ****************************
```

Supplementary Figure 11| Sequence alignment of the promoter region of *pfmdr7*. The sequences of the promoter regions of *pfmdr7* of strains 3D7 (upper) and MEF1 (lower) were aligned using the clustal w. The mutations and deletions are indicted by red.

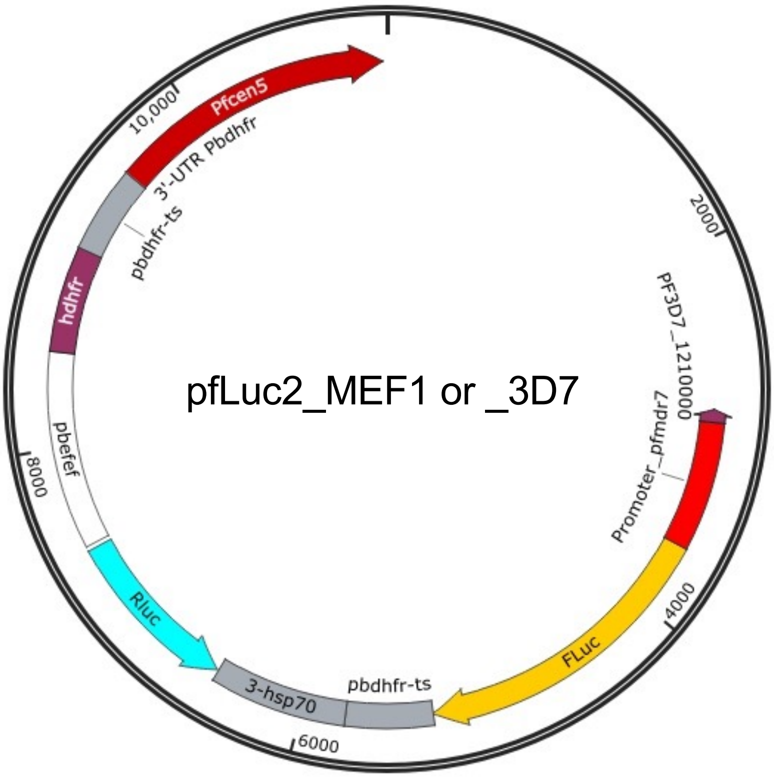

Supplementary Figure 12| Plasmid map of pfLuc2_pfmdr7. The promoter regions of *pfmdr7* of the MEF1 and the 3D7 were cloned upstream of the firefly luciferase gene, indicated as "FLuc". The sea pansy luciferase (RLuc) gene was transcribed using the promoter of elongation factor 1α of *P. berghei*, and was used as an internal control.

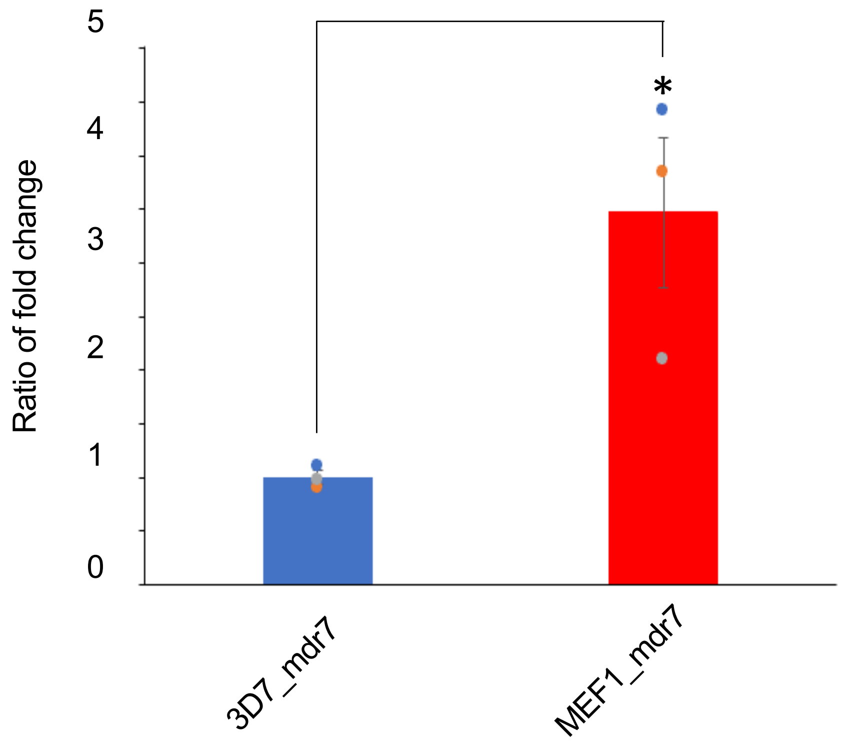

Supplementary Figure 13| quantitative analysis of expression of *pfmdr7* of transgenic parasites. The mRNA were purified from the transgenic parasites, in which the *pfmdr7* of strains 3D7 (3D7_mdr7, blue) and MEF1 (MEF1_mdr7, red) were introduced using the pFCENv1. The mRNA level of *pfmdr7* in those transgenic parasites were analyzed by RT-qPCR in biologically independent triple assays. The relative change of the *pfmdr7* expression in those transgenic parasites were estimated based on that in the 3D7. Error bars are SEM, and measure of the center is the means value. The P-values were calculated from statistical analysis with two-sided Student's t-test. Asterisk indicates P-value (0.024) < 0.05 for comparison between the samples. Source data are provided as a Source Data file.

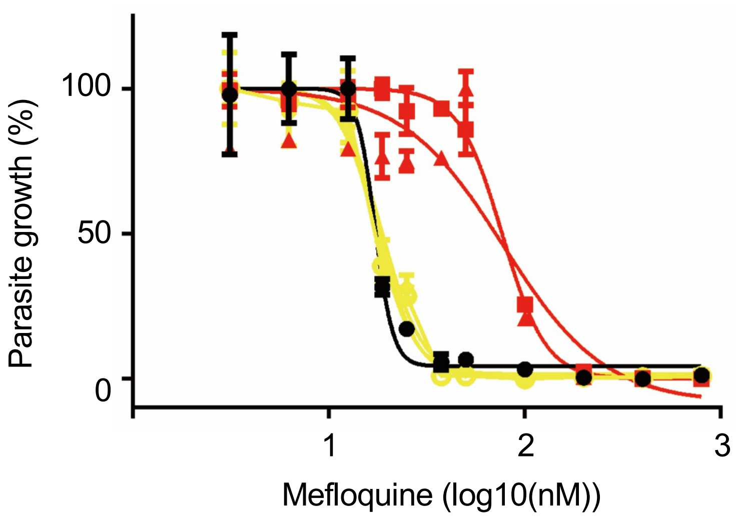

Supplementary Figure 14| Evaluation of the mefloquine resistance of field-isolated parasites, which were used for expression analysis of PfMDR7 mRNA. The IC_{50} values of two mefloquine-resistant (red, MEF18 and 24) and three sensitive (yellow, MEF19, 25, and 26) field-isolated parasites were determined from the obtained curves. All values are presented as the means ± SEs. The IC_{50} values of each parasite were calculated as follows: MEF18, 77.9 nM; MEF24, 76.6 nM; MEF19, 18.3 nM; MEF25, 17.6 nM; MEF26, 18.7 nM. In addition, strain 3D7 (black) was used as a negative control, and its IC_{50} value was 17.2 nM in this assay. SEM were indicated as error bars. All assay were performed using biologically independent 4 samples. Source data are provided as a Source Data file.

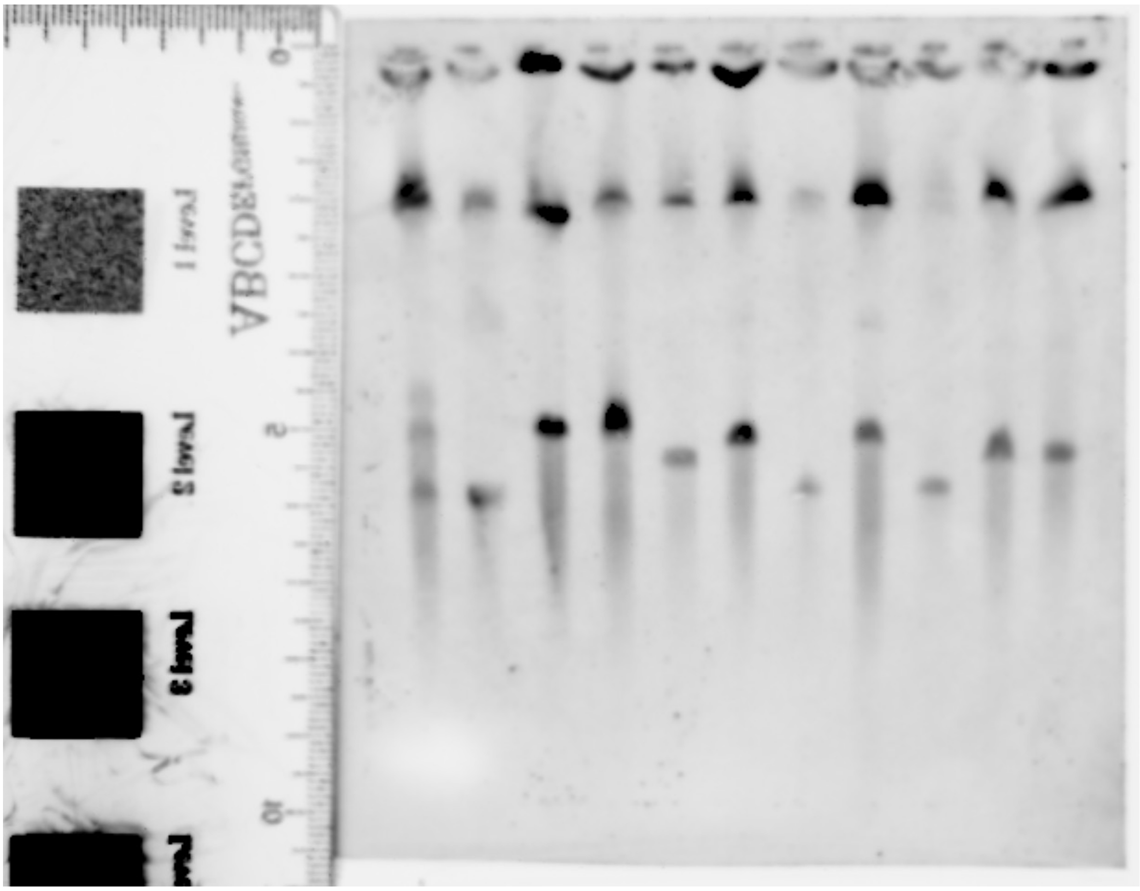

Original image of Supplementary Figure 1c
lane 1: pooled library
lane2-11: parasite clones obtained by limiting dilution
