# Supplementary material for: Genome-wide functional screening of drug-resistance genes in Plasmodium falciparum
Source: Nat Commun. 2022 Oct 18;13:6163. doi: 10.1038/s41467-022-33804-w (PMC9579134; doi:10.1038/s41467-022-33804-w)
Supplement: Supplementary file 3 — Description of Additional Supplementary Files [file 41467_2022_33804_MOESM3_ESM.pdf]

Description of Additional Supplementary Files

File Name: Supplementary Data 1
Description: Copy number variation analysis of ABC transporters in the strain MEF1 was performed based on whole-genome sequencing data using Ion-proton System and the read-depth approach. The obtained reads were mapped on the genomic sequence and the gene-coding sequence the strain 3D7, which are deposited in PlasmoDB ((http://plasmodb.org/plasmo/). Eight genes, which are indicted in the top table, were used as internal controls. The summary of the CNV analysis of ABC transporters were shown in the bottom table.

File Name: Supplementary Data 2
Description: Gene information of DNA fragments, which were recovered from the selected parasites from genomic libraries of MEF1 strain. The genes identified in insert DNA fragments recovered in the first and second experiments are shown in top and bottom tables, respectively. The *pfmdr7*, of which gene ID is PF3D7_1209900, is indicted by red.

File Name: Supplementary Data 3
Description: The *pfmdr7* genotypes and mefloquine resistances of field-isolated parasites, which were collected from patients living in Thai-Myanmar border region. The second row shows the insertion of three amino acids, Asn-Val-Arg (9bp), at the position 27 compared to pfmdr7 of the 3D7. The third row shows the insertion and deletion of Asn residues at the position 156 compared to *pfmdr7* of the 3D7.
