# Supplementary material for: Genome-wide functional screening of drug-resistance genes in Plasmodium falciparum
Source: Nat Commun. 2022 Oct 18;13:6163. doi: 10.1038/s41467-022-33804-w (PMC9579134; doi:10.1038/s41467-022-33804-w)
Supplement: Supplementary file 7 — Reporting Summary [file 41467_2022_33804_MOESM7_ESM.pdf]

nature portfolio

Corresponding author(s): SHIROH IWANAGA

Last updated by author(s): Sep 7, 2022

Reporting Summary

Nature Portfolio wishes to improve the reproducibility of the work that we publish. This form provides structure for consistency and transparency in reporting. For further information on Nature Portfolio policies, see our Editorial Policies and the Editorial Policy Checklist.

Statistics

For all statistical analyses, confirm that the following items are present in the figure legend, table legend, main text, or Methods section.

n/a	Confirmed	
☐	☒	The exact sample size (*n*) for each experimental group/condition, given as a discrete number and unit of measurement
☐	☒	A statement on whether measurements were taken from distinct samples or whether the same sample was measured repeatedly
☐	☒	The statistical test(s) used AND whether they are one- or two-sided<br>*Only common tests should be described solely by name; describe more complex techniques in the Methods section.*
☐	☒	A description of all covariates tested
☐	☒	A description of any assumptions or corrections, such as tests of normality and adjustment for multiple comparisons
☐	☒	A full description of the statistical parameters including central tendency (e.g. means) or other basic estimates (e.g. regression coefficient) AND variation (e.g. standard deviation) or associated estimates of uncertainty (e.g. confidence intervals)
☐	☒	For null hypothesis testing, the test statistic (e.g. *F*, *t*, *r*) with confidence intervals, effect sizes, degrees of freedom and *P* value noted<br>*Give P values as exact values whenever suitable.*
☒	☐	For Bayesian analysis, information on the choice of priors and Markov chain Monte Carlo settings
☒	☐	For hierarchical and complex designs, identification of the appropriate level for tests and full reporting of outcomes
☒	☐	Estimates of effect sizes (e.g. Cohen's *d*, Pearson's *r*), indicating how they were calculated

Our web collection on statistics for biologists contains articles on many of the points above.

Software and code

Policy information about availability of computer code

Data collection	We did not use any software for data collection.
Data analysis	We used the samtools (version 1.10) for SNPs analysis.

For manuscripts utilizing custom algorithms or software that are central to the research but not yet described in published literature, software must be made available to editors and reviewers. We strongly encourage code deposition in a community repository (e.g. GitHub). See the Nature Portfolio guidelines for submitting code & software for further information.

Data

Policy information about availability of data

All manuscripts must include a data availability statement. This statement should provide the following information, where applicable:

- Accession codes, unique identifiers, or web links for publicly available datasets
- A description of any restrictions on data availability
- For clinical datasets or third party data, please ensure that the statement adheres to our policy

The genomic sequence data for strain MEF1 is deposited at NCBI (https://www.ncbi.nlm.nih.gov/sra/?term=DRA004454, Accession number: DRX049711) . In addition, the genomic sequence of P. falciparum 3D7 version 35, which is deposited in PlasmoDB (https://plasmodb.org/plasmo/app/) , was used as the reference sequence. All infomation about the deposited data and reference sequence were indicated in DATA AVAILABILITY.

Human research participants

Policy information about studies involving human research participants and Sex and Gender in Research.

Reporting on sex and gender	In this study, we used only parasite samples, which were obtained from patients. We used neither sex nor gender information of patients.
Population characteristics	We did not use any information about the patients in this study.
Recruitment	The parasite samples used in this study were collected from the patients, who visited Mae Hong Son general hospital, according to the ethical permission obtained from Chang Mai University. When we collected the parasite sample from patients, we obtained informed consent from them.
Ethics oversight	Ethical clearance for the collection of parasite-infected blood was obtained from the Research Ethics Committees at the Faculty of Medicine, Chiang Mai University (permission number: 187/2554) and from the Department of Medicine, Mie University, Japan (permission number: 1312).

Note that full information on the approval of the study protocol must also be provided in the manuscript.

Field-specific reporting

Please select the one below that is the best fit for your research. If you are not sure, read the appropriate sections before making your selection.

☒ Life sciences ☐ Behavioural & social sciences ☐ Ecological, evolutionary & environmental sciences

For a reference copy of the document with all sections, see nature.com/documents/nr-reporting-summary-flat.pdf

Life sciences study design

All studies must disclose on these points even when the disclosure is negative.

Sample size	All experiments including qPCR, IC50 assay, and reporter assay were performed using 2-4 biological independent samples. In particular, we used at least three biological independent samples when we evaluated the results using two-sided Student t-test. We think the number of biological sample are sufficient to ensure the reproducibility and statical difference of the results.
Data exclusions	We did not exclude any data from the results obtained by experiments.
Replication	We confirmed the reproducibility of the results obtained in at least three replicates in all assays.
Randomization	In this study, there were not any experiment which required the randomization of the samples.
Blinding	In this study, there were not any experiment which required the blinding of the samples.

Reporting for specific materials, systems and methods

We require information from authors about some types of materials, experimental systems and methods used in many studies. Here, indicate whether each material, system or method listed is relevant to your study. If you are not sure if a list item applies to your research, read the appropriate section before selecting a response.

Materials & experimental systems

n/a	Involved in the study	
☒	☐	Antibodies
☒	☐	Eukaryotic cell lines
☒	☐	Palaeontology and archaeology
☐	☒	Animals and other organisms
☒	☐	Clinical data
☒	☐	Dual use research of concern

Methods

n/a	Involved in the study	
☒	☐	ChIP-seq
☒	☐	Flow cytometry
☒	☐	MRI-based neuroimaging

Animals and other research organisms

Policy information about studies involving animals; ARRIVE guidelines recommended for reporting animal research, and Sex and Gender in Research

Laboratory animals	We did not use any laboratory animals in this study.
Wild animals	We did not use any wild animals in this study.
Reporting on sex	We did not use sex information in this study.
Field-collected samples	The parasite samples used in this study were collected from the patients, who visited Mae Hong Son general hospital, according to the ethical permission obtained from Chang Mai University in 2012. When we collected the parasite sample from patients, we obtained informed consent from them.
Ethics oversight	Ethical clearance for the collection of parasite-infected blood was obtained from the Research Ethics Committees at the Faculty of Medicine, Chiang Mai University (permission number: 187/2554) and from the Department of Medicine, Mie University, Japan (permission number: 1312).

Note that full information on the approval of the study protocol must also be provided in the manuscript.
